# SARS‐CoV‐2 and HIV coinfection: clinical experience from Rhode Island, United States

**DOI:** 10.1002/jia2.25573

**Published:** 2020-07-01

**Authors:** Katrina M Byrd, Curt G Beckwith, Joseph M Garland, Jennie E Johnson, Su Aung, Susan Cu‐Uvin, Dimitrios Farmakiotis, Timothy Flanigan, Fizza S Gillani, Raul Macias‐Gil, Maria Mileno, Bharat Ramratnam, Natasha R Rybak, Martha Sanchez, Karen Tashima, Eleftherios Mylonakis, Rami Kantor

**Affiliations:** ^1^ Division of Infectious Diseases Department of Medicine The Brown Alpert Medical School and The Miriam Hospital Providence RI USA

**Keywords:** SARS‐CoV‐2, HIV, COVID‐19, coinfection, preparedness

## Abstract

**Introduction:**

Severe Acute Respiratory Syndrome Coronavirus 2 (SARS‐CoV‐2) has infected >6 million people worldwide since December 2019. Global reports of HIV/SARS‐CoV‐2 coinfection are limited. To better understand the impact of the coronavirus disease 2019 (COVID‐19) pandemic on persons with HIV and improve their care, we present an outpatient and inpatient clinical experience of HIV/SARS‐CoV‐2 coinfection from Rhode Island, US.

**Methods:**

We describe outpatient and inpatient preparedness for the COVID‐19 pandemic, and present a case series of all known patients with HIV/SARS‐CoV‐2 coinfection at The Miriam Hospital and Rhode Island Hospital, and The Miriam Hospital Infectious Diseases and Immunology Center, in Providence, Rhode Island, US.

**Results and discussion:**

The Infectious Diseases and Immunology Center rapidly prepared for outpatient and inpatient care of persons with HIV and SARS‐CoV‐2. Between 30 March and 20 May 2020, 27 patients with HIV were diagnosed with SARS‐CoV‐2. Twenty were male, six female and one transgender female; average age was 49 years; 13/27 were Hispanic and 6/27 were African American. All had HIV viral load <200 copies/mL and were on antiretroviral therapy with CD4 count range 87 to 1441 cells/µL. Twenty‐six of the 27 had common COVID‐19 symptoms for one to twenty‐eight days and most had other co‐morbidities and/or risk factors. Nine of the 27 were hospitalized for one to thirteen days; of those, three lived in a nursing home, six received remdesivir through a clinical trial or emergency use authorization and tolerated it well; eight recovered and one died. Overall, 17% of known Center people had HIV/SARS‐CoV‐2 coinfection, whereas the comparable state‐wide prevalence was 9%.

**Conclusions:**

We highlight challenges of outpatient and inpatient HIV care in the setting of the COVID‐19 pandemic and present the largest detailed case series to date from the United States on HIV/SARS‐CoV‐2 coinfection, adding to limited global reports. The aggregated clinical findings suggest that the clinical presentation and outcomes of COVID‐19 appear consistent with those without HIV. Whether SARS‐CoV‐2 infection is more frequent among persons with HIV remains to be determined. More data are needed as we develop our understanding of how HIV and antiretroviral therapy are affected by or have an impact on this pandemic.

## 1 INTRODUCTION

The first case of coronavirus disease 2019 (COVID‐19), caused by Severe Acute Respiratory Syndrome Coronavirus 2 (SARS‐CoV‐2) was reported in Wuhan, China in December 2019 [1]. Within months, the virus infected more than six million people, causing over 350,000 deaths worldwide. The first COVID‐19 case in the US was diagnosed on January 20, 2020, and as of June 02, the US leads the world in both COVID‐19 cases (1,857,872) and deaths (107,911) [2, 3].

There are 37.9 million persons with HIV (PWH) globally, and 1.1 million in the US [4]. PWH may be considered vulnerable to infections due to CD4 cell depletion. However, despite the large scale of the COVID‐19 pandemic, little is known about its characteristics in PWH. To date, there are only 158 confirmed HIV/SARS‐CoV‐2 coinfections reported in detail in the literature, nine from China [5, 6, 7, 8], 56 from Spain [9, 10], 33 from Germany [11], 47 from Italy [12] and 13 from the US [13, 14] with diverse clinical severity and host characteristics including HIV‐related immunosuppression.

The first Rhode Island (RI) COVID‐19 case was diagnosed on 1 March, and as of 2 June, there were 15,441 cases (1.4% of the RI population) and 772 fatalities (4.9% of cases) [15]. Of 1.06 million people in RI, approximately 2800 (0.3%) had HIV in 2018 [16]. Uniquely, the Miriam Hospital Infectious Diseases and Immunology Center (the Center) cares for more than 80% of PWH who are in care in the state. Given limited knowledge of HIV/SARS‐CoV‐2 coinfection, in RI or elsewhere, and to better understand the impact of the COVID‐19 pandemic on PWH and improve their care, we present our outpatient and inpatient clinical experience.

## 2 METHODS

We first describe processes undertaken to achieve appropriate outpatient and inpatient preparedness for the COVID‐19 pandemic at The Miriam Hospital and RI Hospital, primary hospitals for the largest academic medical center in the state, and the Miriam Hospital Infectious Diseases and Immunology Center, in Providence, RI, US. We then present a case series of all known outpatients and inpatients with HIV/SARS‐CoV‐2 coinfection until the time of this writing. Medical records were reviewed for demographic, clinical, laboratory and outcomes data. The study was approved by, and a consent waiver was obtained from the Institutional Review Board at The Miriam Hospital in Providence, RI.

## 3 RESULTS AND DISCUSSION

### 3.1 Outpatient preparedness

The Center is a large, Ryan White‐funded HIV clinic, caring for more than 1800 patients; 98% meeting statewide income eligibility for financial assistance, and 76% reporting incomes below the federal poverty line. In 2019, 99% of Center patients were prescribed ART and 91% had HIV viral load (VL) <200 copies/mL.

After the first COVID‐19 case in RI, the Center rapidly converted most patient visits to telehealth and deferred nonurgent laboratory testing. Understanding challenges PWH might face during social distancing, and realizing potential health consequences of changing insurance status due to job loss and furloughs, outreach‐worker staff proactively assist patients with insurance and medication access; the hospital pharmacy offers no‐charge home medication delivery; pharmacy liaisons assist with co‐pays; behavioral therapists use telehealth; and social workers address community challenges and evaluate and help to address food insecurities.

In regards to COVID‐19 testing and evaluation, the Center implemented a health‐system‐wide telehealth programme to evaluate suspected ambulatory COVID‐19 cases; completed construction of a negative‐pressure exam room to evaluate suspected walk‐in cases; streamlined testing access; provided post‐hospitalization telehealth visits to patients discharged with COVID‐19 (both with and without HIV); and served as an active site for COVID‐19 clinical trials.

### 3.2 Inpatient preparedness

The Center operates a dedicated inpatient teaching “Immunology Service” (the Service) for admitted Center patients, staffed by Center faculty, with multidisciplinary Center staff coordination. With COVID‐19 hospitalizations, the Service was made aware of all COVID‐19 cases and provided an important link between admitted PWH and available COVID‐19 clinical trials. The Infectious Diseases Division, which encompasses the Service, enrolled COVID‐19 patients into two Phase 3 remdesivir clinical trials (ClinicalTrials.gov, NCT04292730, NCT04292899) and a convalescent plasma therapy study. The Center is also a site for an ambulatory study of azithromycin and hydroxychloroquine. Importantly, PWH were not excluded from these trials and the Service actively referred PWH to them. It is crucial that PWH enroll in such trials, as we offer potential life‐saving treatments, and learn whether HIV itself or HIV‐related immunosuppression predispose PWH to different clinical presentations, courses, or outcomes compared to persons without HIV, and whether ART provides protection from SARS‐CoV‐2.

### 3.3 Case series

Between 30 March and 20 May, 27 RI PWH were diagnosed with COVID‐19 using nucleic acid‐based testing; 0.18% of COVID‐19 diagnoses in the state during that time period. Nine were hospitalized (Table 1) and 18 were outpatients (Table 2). Overall, patients’ HIV was diagnosed between 1980 and 2019; 20 were male, six female and one transgender female, ages 30 to 71 years; 13/27 Hispanic and 6/27 African American; most were diagnosed with COVID‐19 in the outpatient or emergency department setting; and all had HIV VL < 200 copies/mL and were on antiretroviral therapy with a CD4 count range 87 to 1441 cells/µL. Twenty‐six of the 27 had common COVID‐19 symptoms for one to twenty‐eight days and most had other co‐morbidities and/or risk factors, with no apparent differences between hospitalized and non‐hospitalized patients. Chest imaging, if obtained, ranged from normal X‐rays to computerized tomography multifocal airspace disease with ground glass infiltrates. Abnormal imaging findings were more common among hospitalized patients. The nine hospitalized patients (Table 1) were admitted for one to thirteen (average 8) days. Of those, three lived in nursing‐homes, three had O2 saturations < 94% at presentation, six received remdesivir through a clinical trial or emergency use authorization, and none required intensive care unit admission. Most had COVID‐19‐associated laboratory abnormalities, reflecting lymphopenia and inflammation. Of the hospitalized patients, eight were discharged and one died. This patient had advanced metastatic renal cell carcinoma and goals of care focused on his comfort. Eighteen of the 27 were followed in the outpatient setting and did not require hospitalization (Table 2).

**Table 1:** Inpatient HIV and SARS‐CoV‐2 coinfection in Rhode Island

	Patient 1	Patient 2	Patient 3	Patient 4	Patient 5	Patient 6	Patient 7	Patient 8	Patient 9
General									
Sex (gender)	Male	Male	Male	Male	Male	Male	Male (female)	Male	Female
Age (years)	63	52	63	43	59	45	39	71	60
Race/ethnicity	AA	Hispanic	Hispanic	Hispanic	AA	Hispanic	Hispanic	White (non‐Hispanic)	White (non‐Hispanic
HIV									
Dx year	1989	2005	2011	2010	2002	2009	2005	1999	1980’s
Current ART	ABC/DTG/3TC	EVG/c/FTC/TAF	EVG/c/FTC/TAF	EVG/c/FTC/TAF	EFV, ABC	EFV/FTC/TDF	BTG/FTC/TAF	ABC/DTG/3TC	BTG/FTC/TAF
VL (copies/mL)^a^	<20	<20	<20	<20	<20	<20	<20	<20	<20
CD4 count^a^ (cells/µL)	442	567	87	700	936	334	835	361	1189
CD4% ^a^	13	31	9	42	42	23	33	45	56
Time CD4> 200	Since 2011	Since 2005	Never	Since 2011	Since 2002	Since 2010	Since 2008	Since 2003	At least since 2008
COVID‐19									
Risk factors	Nursing home with + cases	None	None	Recent travel	Nursing home with + cases	Contact with + cases	Contact with + cases	Nursing home with + cases	Recent hospitalization
Other Co‐morbidities	Dementia, CVA	Obesity, HTN	DM, HLD	None	Cancer, ESRD	None	Obesity	Alcoholism	COPD, Cocaine use, DM
Active tobacco use	No (former smoker)	No	No (former smoker)	Yes	No	No	No	Yes	Yes
Symptoms	SOB, lethargy	SOB, fever, HA	SOB	SOB, cough, sore throat, chest pain	SOB	SOB, HA	SOB, fever, HA	Lethargy, decreased appetite	SOB, fever, chills
Symptoms pre‐testing	1 day	8 days	28 days	14 days	1 day	8 days	7 days	7 days	2 days
Min RA O2%	70%	86%	79%	95%	94%	94%	94%	95%	85%
ALC (10^9^/L)^b^	300	600	1300	1370	500	500	1000	600	600
CRP (mg/L)^c^	202	102	25	4	N/A	276	296	209	134
Ferritin (ng/mL)^c^	163	3179	500	321	N/A	1095	1600	804	159
D‐dimer(ng/mL)^c^	N/A	514	245	252	N/A	255	189	747	N/A
ALT (IU/L)^c^	72	102	62	29	N/A	113	116	3	21
AST IU/L)^c^	81	76	45	31	N/A	103	50	18	25
LDH (IU/L)^c^	154	337	236	252	N/A	468	367	147	250
HCV status	Cured	Neg	Neg	Neg	Neg	Cleared	Neg	Neg	Cured
Imaging	CXR: bibasilar asd	CXR: bilateral asd	CXR: bilateral asd	CT mf ground glass infiltrates	CXR: diffuse BL asd	CXR: mf BL asd	CXR: BL asd	CXR: right asd	CXR: emphysematous changes
COVID‐19 therapy	RDV (10 days)	RDV (8 days)	RDV (10 days)	No	No	No	RDV (4 days)	RDV (2 days)	RDV (5 days)
Time in hospital	13 days	8 days	13 days	6 days	8 days	1 day	8 days	4 days	8 days
Outcome	DC	DC	DC	DC	Died	DC	DC	DC	DC

/c, cobicistat; +, positive; 3TC, lamivudine; AA, African American; ABC, abacavir; ALC, absolute lymphocyte count; ART, antiretroviral therapy; asd, air space disease; BL, bilateral; BTG, bictegravir; CD4 count/VL are prior to SARS‐CoV‐2 infection; cong, congestion; CT, computerized tomography; CVA, cerebrovascular accident; CXR, chest X‐ray; DC, discharge; DM, diabetes mellitus, DRV, darunavir; DTG, dolutegravir; dx, diagnosis; ED, emergency department; EFV, efavirenz; ESRD, end stage renal disease; EVG, elvitegravir; FTC, emtricitabine; HA, headache; HCV, hepatitis C virus; HLD, hyperlipidaemia; HTN, hypertension; ICU, intensive care unit; IST, interstitial; mf, multifocal; Min, minimal; N/A, not available; Neg, negative; O2, oxygen; RA, room air; RDV, remdesivir; RPV, rilpivirine; RUL, right upper lobe; Sat, saturation; SOB, shortness of breath; TAF, tenofovir alafenamide; TDF, tenofovir disoproxil fumarate, VL, viral load.

^a^ Before COVID‐19

^b^ lowest recorded

^c^ highest recorded.

**Table 2:** Outpatient HIV and SARS‐CoV‐2 coinfection in Rhode Island

	Pt 1	Pt 2	Pt 3	Pt 4	Pt 5	Pt 6	Pt 7	Pt 8	Pt 9	Pt 10	Pt 11	Pt 12	Pt 13	Pt 14	Pt 15	Pt 16	Pt 17	Pt 18
General																		
Sex	Male	Male	Female	Female	Male	Male	Male	Male	Female	Male	Male	Male	Male	Male	Male	Male	Female	Female
Age (years)	62	37	57	51	32	53	48	38	37	57	56	44	54	37	30	42	47	51
Race/ethnicity	White; non‐Hispanic	White; non‐Hispanic	Other	Hispanic	Hispanic	White; non‐Hispanic	AA	White; non‐Hispanic	AA	Hispanic	Hispanic	Hispanic	Hispanic	Hispanic	White; non‐Hispanic	Hispanic	AA	AA
HIV																		
Dx year	2015	2012	2015	2001	2012	2009	2002	2013	2015	2017	2001	2014	2010	2010	2019	2005	2009	2017
Current ART	BTG/FTC/TAF	BTG/FTC/TAF	BTG/FTC/TAF	DTG/RPV	BTG/FTC/TAF	EVG/c/FTC/TAF	EVG/c/FTC/TAF	EVG/c/FTC/TAF	EVG/c/FTC/TAF	BTG/FTC/TAF	BTG/FTC/TAF	BTG/FTC/TAF	BTG/FTC/TAF	BTG/FTC/TAF	BTG/FTC/TAF	BTG/FTC/TAF	DVR/c/FTC/TAF	BTG/FTC/TAF
VL (copies/mL)^a^	<20	69	<20	<20	<20	<20	46	<20	<20	<20	<20	46	<20	<20	<20	<20	<20	<20
CD4 count ^a^ (cells/µL)	746	789	216	746	346	683	1375	760	644	1441	444	357	813	638	459	743	347	239
CD4% ^a^	23	35	16	21	19	34	35	30	27	53	27	14	36	32	36	18	32	16
Time CD4> 200	Since 2015	Since 2017	Since 2015	Since 2012	Since 2016	Since 2010	Since 2003	Since 2016	Since 2015	Since 2017	Since 2002	Since 2016	Since 2010	Since 2011	Since 2019	Since 2006	Since 2009	Since 2017
COVID‐19																		
Risk factors	Works in homeless Shelter	Contact with + cases	Contact with + cases	None	Contact with + cases	Contact with + cases; travel	Contact with + cases	Healthcare worker	None	None	Contact with + cases	Healthcare worker	Contact with + cases	Contact with + cases	None	None	Works in group home	Healthcare worker
Other Co‐morbidities	HTN	Obesity	DM	DM, asthma	HLH in remission, Histo‐plasmosis	HTN	HTN	Obesity	None	None	HTN, obesity	Obesity	HTN, CAD	Alcoholism, cirrhosis	None	None	None	Obesity
Active tobacco use	No	No (former smoker)	No	No (former smoker)	No (former smoker)	Yes	No	No (former smoker)	No	No	No	No (former smoker)	No	No (former smoker)	Yes	No	No	No
Symptoms	Cough, fever, low appetite	Cough, fever, myalgia	None	Fever, myalgia, HA, chills	Fever, cough, sore throat	Anosmia, nasal cong	Fatigue, nasal cong, HA	Myalgia, back pain	HA	HA, myalgia, anosmia	HA, myalgia, anosmia	Myalgia, back pain	Lethargy, anosmia	SOB, fever, myalgia	Anosmia	Cough, myalgia	Fever, cough	HA, back pain
Symptoms pre‐testing	14 days	2 days	N/A	1 day	3 days	weeks	4 days	3 days	Unknown	7 days	Unknown	5 days	Unknown	Unknown	Unknown	9 days	Unknown	4 days
Min RA O2%	96%	98%	N/A	98%	N/A	N/A	N/A	N/A	N/A	97%	N/A	95%	N/A	95%	N/A	N/A	N/A	99%
ALC (10^9^/L)^b^	2200	800	N/A	1800	N/A	N/A	N/A	N/A	N/A	1400	N/A	2200	N/A	2100	N/A	N/A	N/A	1600
CRP (mg/L)^c^	N/A	N/A	N/A	N/A	N/A	N/A	N/A	N/A	N/A	180	N/A	N/A	N/A	N/A	N/A	N/A	N/A	N/A
Ferritin (ng/mL)^c^	N/A	N/A	N/A	N/A	N/A	N/A	N/A	N/A	N/A	412	N/A	N/A	N/A	N/A	N/A	N/A	N/A	N/A
D‐dimer (ng/mL)^c^	N/A	N/A	N/A	N/A	N/A	N/A	N/A	N/A	N/A	142	N/A	N/A	N/A	N/A	N/A	N/A	N/A	N/A
ALT (IU/L)^c^	N/A	14	N/A	25	N/A	N/A	N/A	N/A	N/A	24	N/A	N/A	N/A	61	N/A	N/A	N/A	10
AST IU/L)^c^	N/A	31	N/A	21	N/A	N/A	N/A	N/A	N/A	30	N/A	N/A	N/A	71	N/A	N/A	N/A	18
LDH (IU/L)^c^	N/A	N/A	N/A	N/A	N/A	N/A	N/A	N/A	N/A	245	N/A	N/A	N/A	N/A	N/A	N/A	N/A	N/A
HCV status	Neg	Neg	Neg	Neg	Neg	Neg	Neg	Neg	Neg	Neg	Neg	Neg	Neg	Neg	Neg	Neg	Cured	Neg
Imaging	CXR: mild ist asd	CXR: clear lungs	N/A	CXR: RUL asd	N/A	N/A	N/A	N/A	N/A	CXR: BL asd	N/A	CXR: BL asd	N/A	CXR: ist asd	N/A	N/A	N/A	Neg
COVID‐19 therapy	No	No	No	No	No	No	No	No	No	No	No	No	No	No	No	No	No	No
Outcome	DC from ED	DC from ED	Home	DC from ED	Home	Home	Home	Home	Home	Home	Home	DC from ED	Home	Home	Home	Home	Home	Home

/c, cobicistat; +, positive; 3TC, lamivudine; AA, African American; ABC, abacavir; ALC, absolute lymphocyte count; ART, antiretroviral therapy; asd, air space disease; BL, bilateral; BTG, bictegravir; CD4 count/ VL are prior to COVID‐19 infection; cong, congestion; CT, computerized tomography; CVA, cerebrovascular accident; CXR, chest X‐ray; DC, discharge; DM, diabetes mellitus, DRV, darunavir; DTG, dolutegravir; dx, diagnosis; ED, emergency department; EFV, efavirenz; ESRD, end stage renal disease; EVG, elvitegravir; FTC, emtricitabine; HA, headache; HCV, hepatitis C virus; HLD, hyperlipidaemia; HLH, haemophagocytic lymphohistiocytosis; HTN, hypertension; ICU, intensive care unit; IST, interstitial; mf, multifocal; Min, minimal; N/A, not available; Neg, negative; O2, oxygen; Pt, patient; RA, room air; RDV, remdesivir; RPV, rilpivirine; RUL, right upper lobe; Sat, saturation; SOB, shortness of breath; TAF, tenofovir alafenamide; TDF, tenofovir disoproxil fumarate, VL, viral load.

^a^ Before COVID‐19

^b^ lowest recorded

^c^ highest recorded.

### 3.4 Case series highlights

We highlight several unique points related to these 27 PWH and COVID‐19. First, the detailed description of the 27 RI patients adds to the limited HIV/SARS‐CoV‐2 coinfection literature. Patients had typical COVID‐19 symptoms like cough, shortness of breath, lethargy, fever, headache, sore throat, chest pain, myalgia and anosmia. Twenty were male, six female and one transgender female, most Hispanic or African American (including the one patient who died), with an average age 49 years. More than half recovered at home without requiring hospitalization. HIV VL was <200 copies/mL for all, and with the exception of hospitalized patient #3, all had CD4 count >200 cells/µL. Taken together, despite concerns for higher COVID‐19 prevalence in PWH with CD4 counts <200 or potentially poorer overall clinical outcomes in PWH, our data are consistent with reports from outside the US, suggesting that COVID‐19 is not more frequent among virologically suppressed PWH on ART. Additionally, clinical presentations, course and outcomes appear consistent with persons without HIV. It is still too early to draw any definitive conclusions from these few cases, and additional larger studies are needed.

Second, three patients, all hospitalized, lived in nursing‐homes with other residents with COVID‐19. All were initially asymptomatic; patient #1 became acutely confused and hypoxic leading to hospitalization; patient #5 was admitted for pain control in the setting of metastatic cancer, developed hypoxia and tachypnoea on hospital day 8, when he opted for comfort care and subsequently died; and patient #8 was admitted for the complication of decreased oral intake and altered mental status. These cases highlight that nursing‐home residents were vulnerable to COVID‐19, likely because they were typically older, have co‐morbidities, live in close proximity to other at‐risk persons, and have prolonged interactions with caregivers, as described [17]. Many reported individuals in these settings were asymptomatic at testing, as were our three patients, suggesting asymptomatic infection, with or without HIV [17].

Third, we report the first six PWH and COVID‐19 in RI, adding to the two reported cases from Italy [12] and Spain [10], who received remdesivir. All six tolerated it well, including two with history of hepatitis C virus. In a preliminary report of a double‐blind, randomized, placebo‐controlled trial, remdesivir was recently shown to be superior to placebo in shortening the time to recovery in adults hospitalized with COVID‐19 and evidence of lower respiratory tract infection [18]. We eagerly await the final results of ongoing clinical trials evaluating the safety and efficacy of remdesivir for COVID‐19, and it is essential and encouraging that PWH were included in these trials.

Fourth, whether and how HIV antiretrovirals impact acquisition or treatment of COVID‐19 remains unknown. Clinical trials to explore these possibilities are ongoing, for drugs such as tenofovir/emtricitabine, lopinavir/ritonavir and darunavir/cobicistat [19, 20, 21]. A randomized, controlled, open‐label trial involving hospitalized adult patients with severe COVID‐19 showed no benefit of lopinavir‐ritonavir treatment beyond standard of care [22]. All 27 PWH in this series were on antiretroviral therapy; 25/27 on emtricitabine or lamivudine; and 17/18 (94%) of outpatients and 6/9 (67%) of inpatients (*p* = 0.09; Fisher’s Exact Test) on tenofovir formulations; suggesting that these antiretrovirals were not completely protective against SARS‐CoV‐2 infection. Whether ART medications have any impact on infection or outcomes remains to be determined.

Fifth, one RI patient with a CD4 count of 87 cells/µL presented with four weeks of progressive dyspnoea. He underwent bronchoscopy that ruled out *Pneumocystis jirovecii* pneumonia during a separate hospitalization four weeks before his COVID‐19 diagnosis. At that time, COVID‐19 was uncommon and he was not tested. Interestingly, a specimen from that bronchoscopy, subsequently tested, was positive for SARS‐CoV‐2. The patient had a prolonged 28‐day period before re‐hospitalization with respiratory symptoms and O2 saturations down to 79%, and his nasopharyngeal PCR testing remained positive at 38 days, which is on the longer end of the reported SARS‐CoV‐2 shedding range of eight to thirty‐seven days [1]. Whether this prolonged symptomatology and PCR positivity is related to HIV infection, low CD4 count, ART, and/or other factors remains to be determined.

Lastly, with RIs aggressive rollout of ambulatory SARS‐CoV‐2 diagnostic testing, 162 Center patients were tested at the time data collection was completed, of whom the above 27 (17%) were found to be positive; Although this is currently higher than the statewide positivity rate (15,441 /170,739; 9%; *p* = 0.00002 chi square test) [15], these numbers are subject to bias and should be interpreted cautiously, and broader testing, including of asymptomatic individuals and antibody testing, is still urgently needed.

## 4 CONCLUSIONS

We present a RI perspective, to our knowledge the largest detailed case series to date from the US, on HIV/SARS‐CoV‐2 coinfection. Despite being limited by a retrospective study design, small number of cases, limited testing early on and some unavailable data in times of telemedicine, this case series adds to limited global reports. Data are as yet inconclusive on the effect of the COVID‐19 pandemic on PWH [9, 10, 11, 12, 13, 14, 23, 24]. More data on prevalence, clinical characteristics and outcomes of HIV/SARS‐CoV‐2 coinfection are needed to understand the existence of any differences between PWH and persons without HIV. Current guidance advises HIV providers on follow‐up (e.g. telehealth), practice management (e.g. staff support and triage) and medication needs (e.g. 90‐day supply) [25]. These, as well as increased surveillance, testing and continued clinical follow‐up are critical as we develop our understanding of how HIV and antiretroviral therapy are affected by or have an impact on the COVID‐19 pandemic.

## COMPETING INTEREST

All authors declare we have no competing interests.

## AUTHORS’ CONTRIBUTIONS

RK conceived the study and was involved in all its aspects. KB, JMG, EM, FSG and RK compiled the case series. KMB collected all data and wrote the first draft of the manuscript with RK, with contributions from CGB and JMG. All authors read, critically revised and approved the final manuscript.
